# A comparison of oxytocin and carboprost tromethamine in the prevention of postpartum hemorrhage in high-risk patients undergoing cesarean delivery

**DOI:** 10.3892/etm.2013.1379

**Published:** 2013-11-01

**Authors:** JING BAI, QIAN SUN, HUI ZHAI

**Affiliations:** Department of Gynecology and Obstetrics, Jinan Maternity and Child Care Hospital, Jinan, Shandong 250001, P.R. China

**Keywords:** carboprost, oxytocin, postpartum hemorrhage, third stage of labor

## Abstract

The aim of this study was to compare carboprost with oxytocin for the prevention of postpartum hemorrhage (PPH) in females with a high risk of PPH undergoing cesarean delivery. Patients were randomly divided into three groups that received different uterotonics (oxytocin, carboprost and oxytocin plus carboprost) during cesarean section, following the delivery of the infant. A total of 117 females (age range, 19–40 years) at 35–40 weeks gestation who delivered by cesarean between December, 2010 and May, 2012 were included in this study. There were 29 cases of twins, 12 cases of polyhydramnios, 23 cases of placenta previa and 53 cases of fetal macrosomia. There were 37 patients in the oxytocin group, 36 in the carboprost group and 44 in the oxytocin plus carboprost group. No significant differences were identified in maternal age, gravidity/parity, gestational age and reason for cesarean delivery between the three groups. The median blood loss in the oxytocin, carboprost and oxytocin plus carboprost groups was 610, 438 and 520 ml, respectively. The blood loss in the carboprost group was significantly lower than that in the oxytocin and oxytocin plus carboprost groups (both P<0.05). Vomiting occurred in eight patients from the carboprost group, two from the oxytocin group and two from the oxytocin plus carboprost group (P=0.036). Carboprost was more effective than oxytocin in preventing PPH in high-risk patients undergoing cesarean delivery.

## Introduction

Postpartum hemorrhage (PPH) refers to >500 ml blood loss within 24 h following vaginal delivery, >1,000 ml following cesarean delivery, or the requirement for a blood transfusion within 24 h of delivery (1,2). PPH is reported to occur in ~5% of all deliveries, and the risk is significantly greater with cesarean delivery than vaginal delivery (3,4). In China, PPH is the most common serious obstetric complication and the leading cause of maternal mortality, accounting for 49.9% of maternal deaths (5). The leading cause of PPH is uterine atony, followed by retained placenta and injury to the genital tract (1,3). Risk factors for PPH include fetal macrosomy, prolonged labor, multiple pregnancies, polyhydramnios, uterine myoma, placenta previa, grand multiparity and uterine infection (1,3).

The majority of maternal deaths due to PPH may be avoided, and the key lies in early diagnosis and proper treatment. However, PPH is one of the most challenging complications faced by clinicians. Active management of the third stage of labor includes the administration of uterotonic agents following the cesarean section or during the third stage of labor for vaginal delivery, and studies have shown that it may reduce the incidence of PPH (1,6–9). Oxytocin is the most commonly used uterotonic agent for the prevention of PPH, and has been demonstrated to reduce blood loss following delivery (1,8). However, oxytocin has a half-life of <10 min and thus must be administered by continuous intravenous infusion (10). Furthermore, saturation of uterine receptors may occur, and excessive dosages are capable of producing water toxicity due to its antidiuretic effect (10). Other uterotonic agents have been studied, and have been shown to reduce PPH, including carbetocin, a long-acting synthetic oxytocin analogue, ergot alkaloids (such as ergonovine, syntometrine) and prostaglandins (such as misoprostol and carboprost) (11–17).

Carboprost tromethamine (Hemabate) is the synthetic 15-methyl analogue of prostaglandin F_2α_, and has been reported to be 84–96% effective in the treatment of persistent hemorrhage due to uterine atony (18). However, since its introduction, there have been few studies of its effectiveness for the prevention and treatment of PPH, and only one specifically examining its use following cesarean delivery (16,17,19–21).

The purpose of this study was to compare carboprost with oxytocin for the prevention of PPH in high-risk females undergoing cesarean delivery.

## Patients and methods

### Patients

Pregnant females at a high risk of PPH who were scheduled to undergo a cesarean section at the Jinan Maternity and Child Care Hospital (Jinan, China) between December, 2010 and May, 2012 were included in this study. Patients with coagulation disorders or contraindications for receiving prostaglandin drugs were excluded from the study. The patients were randomly divided into three groups and received different uterotonics (oxytocin, carboprost and oxytocin plus carboprost) during cesarean section, following the delivery of the infant. This study was approved by the Institutional Review Board of the Jinan Maternity and Child Care Hospital, and all patients provided written informed consent for participation in the study.

### Interventions

In the oxytocin group, patients received a myometrial injection of 20 units oxytocin (Shanghai Harvest Pharmaceutical Co., Ltd., Shanghai, China) during the cesarean section, immediately following the delivery of the infant, and subsequently a continuous intravenous infusion of 20 units oxytocin diluted in 1,000 ml saline or Ringer’s solution. In the carboprost group, patients received a myometrial injection of 0.25 mg carboprost tromethamine (Pharmacia & Upjohn Company, Kalamazoo, MI, USA) during the cesarean section, immediately following the delivery of the infant. The injection was repeated every 15 min, as required, until a maximum total dose of 2 mg had been administered. In the oxytocin plus carboprost tromethamine group, patients received a myometrial injection of 0.25 mg carboprost tromethamine and continuous intravenous infusion of 20 units oxytocin diluted in 1,000 ml saline or Ringer’s solution during the cesarean section, immediately following the delivery of the infant.

In cases in which uterine bleeding was not able to be effectively controlled by the aforementioned methods and the blood loss was ≥1,000 ml, other methods of control, including the administration of additional uterotonic agents, uterine artery ligation, uterine gauze packing or blood transfusions, were used.

Data recorded and compared included the volume of blood lost during surgery and within 2 h of surgery, preoperative and postoperative hemoglobin levels, and the other methods used to control bleeding, if required.

### Statistical analysis

Continuous variables are presented as the mean ± standard deviation or median with range, depending on the normality of the data distribution. Categorical variables are expressed by frequencies and percentages. The differences among the three groups were detected using analysis of variance (ANOVA) or a Kruskal-Wallis test for continuous variables, and using a Fisher’s exact test for categorical variables, as appropriate. In addition, the differences prior to and following delivery were examined using the paired Student’s t-test or Wilcoxon signed ranks test. For all analyses, a two-sided P<0.05 was considered to indicate a statistically significant difference. Statistical analyses were performed using SPSS 15.0 statistical software (SPSS Inc., Chicago, IL, USA).

## Results

### Patients

A total of 117 females at increased risk of PPH who received a cesarean delivery between December, 2010 and May, 2012 were included in this study. The patients were between 19 and 40 years of age and 35 and 40 weeks gestation. There were 29 cases of twins, 12 cases of polyhydramnios, 23 cases of placenta previa and 53 cases of fetal macrosomia. There were 37 patients in the oxytocin group, 36 in the carboprost group and 44 in the oxytocin plus carboprost group. No significant differences were identified in maternal age, gravidity and parity, gestational age at delivery and reason for cesarean delivery among the three groups (all P>0.05, Table I).

### Blood loss and interventions

Blood loss was ≥1,000 ml in 13 patients from the oxytocin group, four patients from the carboprost group, and three patients from the oxytocin plus carboprost group. In the oxytocin group, the oxytocin dose was increased or other drugs were also administered in 12 cases, bilateral uterine artery ligation was performed in six cases, uterine gauze packing was adopted in two cases and a blood transfusion was performed in four cases (data not shown). In the carboprost group the dosage was increased or other drugs were also administered in three cases, bilateral uterine artery ligation was performed in three cases and uterine gauze packing was adopted in two cases. In the oxytocin plus carboprost group, the carboprost dose was increased or other drugs were also administered in two cases, bilateral uterine artery ligation was performed in two cases and uterine gauze packing was adopted in one case.

**Figure 1:**
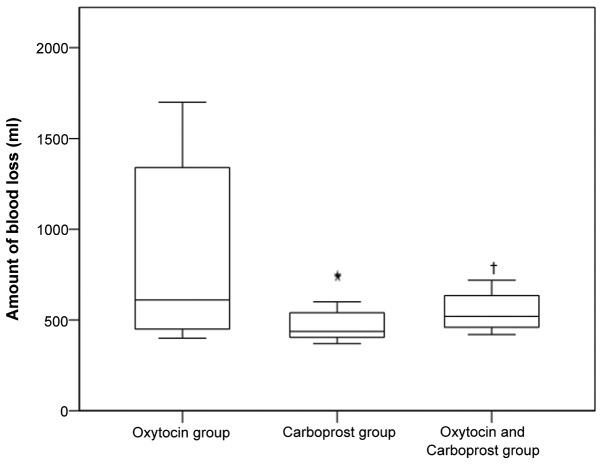
Primary outcome: Amount of blood loss. ^*^Indicates a significant difference between the given group and the oxytocin group. ^†^Indicates a significant difference between the given group and the carboprost group.

**Figure 2:**
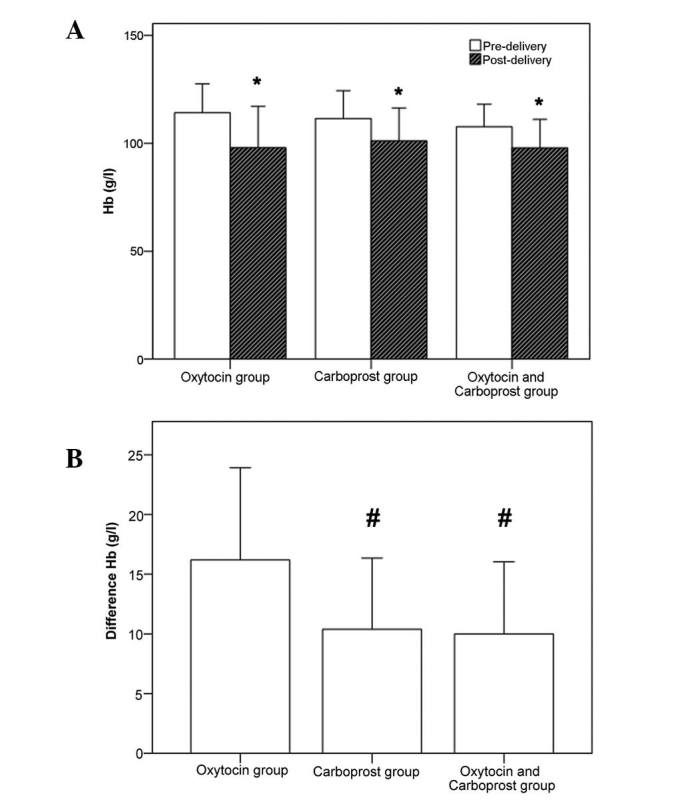
Secondary outcome: Difference in hemoglobin levels prior to and following delivery. (A) Pre- and post-delivery hemoglobin levels for oxytocin, carboprost, and combination of oxytocin and carboprost groups, separately. (B) Difference of hemoglobin levels from pre-delivery to post-delivery for oxytocin, carboprost, and combination of oxytocin and carboprost groups, separately. ^*^Indicates a significant difference prior to and following delivery in each group.^#^Indicates a significant difference between the given group and the oxytocin group.

The median blood loss in the oxytocin, carboprost, and oxytocin plus carboprost groups was 610, 438 and 520 ml, respectively (Fig. 1). The median blood loss in the carboprost group was significantly lower than that in the oxytocin and oxytocin plus carboprost groups (both P<0.05). The change in hemoglobin levels prior to and following delivery in the three groups is shown in Fig. 2. In all three groups, the hemoglobin level following delivery was significantly lower than it had been prior to delivery (all P<0.01, Fig. 2A). Furthermore, there was a significant difference in the reduction in hemoglobin levels among the three groups (P<0.001, Fig. 2B). Patients in the carboprost and oxytocin plus carboprost groups had a significantly smaller reduction in hemoglobin levels compared with the oxytocin group.

### Adverse events

The adverse events that occurred in the three groups are summarized in Table II. Nausea and vomiting were the most common adverse events. Vomiting occurred in eight (22.2%) patients in the carboprost group, as compared with two (5.4%) in the oxytocin group and two (4.5%) in the oxytocin plus carboprost group, and the difference was statistically significant (P=0.036).

## Discussion

The results of the present study demonstrated that carboprost was more effective at preventing PPH than oxytocin in patients at a high risk of PPH undergoing cesarean delivery. The side-effects observed in the three groups were similar, with the exception of vomiting, which was more common in the patients who received carboprost.

To date, PPH remains a leading cause of maternal morbidity and mortality in China, as well as in many underdeveloped countries (2,5). The main causes of PPH are uterine atony, residual trophoblastic tissue, genital tract trauma and clotting disorders. Of these, uterine atony is the most common and is apparent in 70–90% of all cases of PPH (1,2). PPH within 2 h of delivery accounts for ~90% of the total number of cases (1,2).

Oxytocin is the most commonly used drug for the prevention and treatment of excessive bleeding following delivery (8). The most significant benefit of oxytocin is rapid action without causing elevated blood pressure or tetanic uterine contractions. Studies have demonstrated that the routine prophylactic use of oxytocin may reduce the need for additional uterotonics (8,22). However, the use of oxytocin is limited by the dose. Myometrial oxytocin receptor saturation may affect its effectiveness, and excessive dosages may result in coronary artery contraction, hypotension and antidiuretic effect-induced water intoxication (8,23). Therefore, other uterotonics may be required in patients at high-risk of PPH. While it is clear that uterotonics are capable of reducing blood loss during the third stage of labor and preventing PPH, the most effective uterotonic in in the case of cesarean versus vaginal delivery and in certain other circumstances has not been elucidated (1,7).

Carboprost tromethamine is the synthetic 15-methyl analogue of prostaglandin F_2α_. It may be administered via intramuscular injection at a dose of 0.25 mg, and may be repeated every 15 min until a maximum total dose of 2 mg has been administered (24). Carboprost has been reported to be 84–96% effective in the treatment of persistent hemorrhage due to uterine atony, and may avoid the need for surgical intervention (18). However, few studies have examined its use for the prevention of PPH. Vaid *et al*(16) compared prophylactic sublingual misoprostol, intramuscular methylergometrine and intramuscular carboprost for the active management of the third stage of labor, and observed that the three drugs were equally effective in the prevention of PPH, although diarrhea was more common in the patients who received carboprost. In a similar study, Abdel-Aleem *et al*(19) compared carboprost and methylergometrine in 150 females who were randomly assigned to receive one of the two drugs, and observed that the duration of the third stage of labor and mean blood loss were significantly less in the carboprost group. Oleen and Mariano (20) reported that carboprost effectively controlled bleeding in 208 out of 237 (87.8%) cases of PPH.

Carboprost may cause prostaglandin-like side-effects, including nausea, vomiting, diarrhea, headaches, hypertension and bronchial asthma caused by the contraction of smooth muscles (24). It may also act on the thermoregulatory center, increasing the basal body temperature (24). Patients may experience hot flashes, sweating and increased irritability. Lamont *et al*(17) compared carboprost and syntometrine for the prevention of PPH and revealed that although the two drugs were as effective in the prevention of PPH, diarrhea occurred in 21% of the patients who received carboprost, compared with only 0.8% of the patients who received syntometrine. Despite the aforementioned potential side-effects, serious side-effects are rare and self-limited (16). The present results demonstrated that vomiting was relatively common in the patients who received carboprost; however, it was readily managed.

There are limitations to the present study that should be considered. This was a single center study with a relatively small study group; thus, the results may not be generalizable to other patient populations.

In conclusion, the results of this study demonstrated that carboprost was more effective than oxytocin in preventing PPH in high-risk patients undergoing cesarean delivery. The drug was well-tolerated with minimal adverse effects. Carboprost may be considered to be suitable drug for the active management of the third stage of labor in this patient population.

## Figures and Tables

**Table I:** Patient demographic data.

Variables	Oxytocin group (n=37)	Carboprost group (n=36)	Oxytocin + carboprost group (n=44)	P-value
Age (years)^a^	28.65±4.77	27.92±4.29	27.05±3.54	0.230^d^
Reason for cesarean delivery^b^				0.928^e^
Twin pregnancy	11 (29.7)	7 (19.4)	11 (25.0)	
Hydramnios	3 (8.1)	5 (13.9)	4 (9.1)	
Placenta previa	6 (16.2)	7 (19.4)	10 (22.7)	
Macrosomia	17 (45.9)	17 (47.2)	19 (43.2)	
Gestational age (weeks)^a^	37.99±1.22	37.37±1.33	37.99±1.39	0.068^d^
Gravida^c^	2 (1–6)	2 (1–5)	2 (1–4)	0.518^f^
Para^c^	1 (1–3)	1 (1–3)	1 (1–3)	0.696^f^

Data are presented as the ^a^mean ± SD; ^b^number (percentage); and ^c^median (range). P-values are from ^d^analysis of variance (ANOVA); ^e^Fisher’s exact test; and ^f^Kruskal-Wallis test.

**Table II:** Adverse events.

Variables	Oxytocin group (n=37)	Carboprost group (n=36)	Oxytocin + carboprost group (n=44)	P-value
Nausea	2 (5.4)	6 (16.7)	4 (9.1)	0.274
Vomiting	2 (5.4)	8 (22.2)^a^	2 (4.5)^b^	0.036^c^
Fever	2 (5.4)	3 (8.3)	0 (0.0)	0.096
Diarrhea	0 (0.0)	1 (2.8)	1 (2.3)	0.758
Headache	0 (0.0)	1 (2.8)	0 (0.0)	0.297
Elevated blood pressure	0 (0.0)	4 (11.1)	2 (4.5)	0.113

Data are presented as number (percentage).

a Indicates a significant difference between the given group and the oxytocin group.

b Indicates a significant difference between the given group and carboprost group.

c Indicates a significant difference among the three groups.

## Abbreviations

PPH: postpartum hemorrhage
